# CATNON conclusion: Closure on concurrent chemotherapy in IDH mutant grade 3 astrocytoma

**DOI:** 10.1093/neuonc/noag035

**Published:** 2026-02-18

**Authors:** David Schiff

**Affiliations:** Division of Neuro-Oncology, University of Virginia School of Medicine, Charlottesville (D.S.)

The recent updated results of the concurrent and adjuvant temozolomide in non-codeleted gliomas (CATNON) study definitively address initial management of grade 3 isocitrate dehydrogenase mutant (IDHmt) astrocytomas and further our knowledge of molecular biomarkers in this entity. CATNON, launched in 2007, was designed to test the benefit of concurrent and post-radiation temozolomide in patients with anaplastic astrocytomas. Although the study’s inception predated the seminal discovery of the role of IDH mutations in glioma, a subsequent amendment incorporated analyses based upon IDH mutational status. The first interim analysis^1^ demonstrated the benefit of 12 post-radiation temozolomide cycles. The second interim analysis, with a median follow-up of 56 months, reported a significant overall survival (OS) benefit in patients with IDHmt tumors receiving adjuvant temozolomide with no additional benefit from adding concurrent temozolomide.^2^ Another report focused on outcomes in enrolled patients with IDH wild-type gliomas meeting the WHO 2021 molecular criteria for diagnosis of glioblastoma.^3^ The newest and final report of the CATNON study,^4^ with 10.9 years median follow-up, concentrates on the outcome of the two-thirds of enrolled patients with IDHmt tumors.

Arguably the most immediately impactful finding is a convincing reiteration of the previously reported finding that, among IDHmt patients receiving adjuvant temozolomide, the addition of concurrent temozolomide is of no benefit. Whereas the earlier report noted an insignificant hazard ratio (HR) of 0.82 for the benefit of adding concurrent to adjuvant temozolomide, the current publication updated the HR of 0.92; moreover, OS favored the omission of concurrent temozolomide (12.5 vs. 11.7 years). In contrast, the median OS of IDHmt patients receiving neither concurrent or adjuvant temozolomide was only 5.1 years, highlighting the large benefit from temozolomide. Median progression-free survival among patients receiving adjuvant temozolomide was 7.0 years, indicating that even among patients with grade 3 IDHmt tumors (three-quarters of whom received salvage chemotherapy), several years of post-progression survival can be anticipated.

A longstanding question has been whether MGMT methylation status in IDHmt tumors has prognostic or predictive value for benefit of temozolomide. CATNON has yielded a clear answer: MGMT methylation status has no prognostic value whether determined with methylation-specific qPCR or methylation arrays. Nor did MGMT methylation predict benefit from addition of temozolomide to radiotherapy. These findings accord with a recently published large retrospective series reporting no predictive or prognostic significance in IDHmt gliomas.^5^

The CATNON data also lay to rest the notion that age > 40 is prognostically unfavorable in grade 3 astrocytomas. Patients aged 40-60 did as well as those younger than 40. As discussed elsewhere,^6^ the previous inclusion into grade II and III gliomas of what we now classify as glioblastomas led to this now discarded conclusion. Although OS in patients > 60 was worse, they comprised less than 3% of the study IDHmt population.

A notable strength of the current manuscript is the molecular analysis linking potential biomarkers to outcome, of particular importance given the limitations of relying on mitotic count to differentiate grade 2 from 3 astrocytomas.^7 ^^,^^8^ Almost all subjects with IDHmt tumors had tissue available for next generation sequencing as well as DNA methylation profiling that provided copy number analysis as well as methylation-based subtyping. In addition to the aforementioned MGMT analyses, this allowed for determination of CDKN2A/B, PIK3CA, PIK3R1, CDK4, CDK6, PDGFRA, PTEN, and RB1 status. While univariable analysis demonstrated associations with OS for homozygous deletion of CDKN2A/B, PDGFRA amplification, CDK4 amplification, PTEN loss of heterozygosity, and total copy number variation load, on multivariable analysis only CDKN2A homozygous deletion, PDGFR amplification, and methylation profiling retained prognostic significance. While CDKN2A/B homozygous deletion has signified grade 4 designation since implementation of WHO 2021, the similarly poor prognosis with PGDFRA amplification seen in 5% of IDHmt CATNON subjects argues for grade 4 ­designation in the next WHO update. The CATNON results confirm the prognostic value of DNA methylation profiling for classification of IDHmt astrocytomas into astrocytoma high-grade IDHmt versus astrocytoma IDHmt; moreover, scores derived from methylation profiling allowed further refinement into three groups with distinct prognoses. The applicability of these results will depend on more widespread implementation of this tool.

The authors suggest that because there was no significant interaction between CDKN2A/B status and outcome from receiving any temozolomide versus radiation alone, their results support radiotherapy followed by 12 cycles of temozolomide as standard of care for IDHmt grade 4 astrocytoma. However, as this conclusion was based on only 44 tumors with CDKN2A/B homozygous deletion, this proposition should be taken with a grain of salt.

An extraordinary amount of knowledge about histologically intermediate grade adult diffuse gliomas has accrued since CATNON’s initial design more than 20 years ago. CATNON’s statistical assumption was that OS with radiation alone would be 24 months, and an improvement with either concurrent or adjuvant temozolomide to 31 months would represent a positive outcome. In fact, with the addition of adjuvant temozolomide to radiotherapy, CATNON found a doubling of OS, to more than a decade, in grade 3 IDHmt astrocytomas. The authors deserve congratulations for incorporating evolving molecular insights and techniques into initially unplanned analyses to maximize knowledge gleaned from this pivotal study.

## Conflict of Interest Statement

None declared.
